# EZH2 suppresses endogenous retroviruses and an interferon response in cancers

**DOI:** 10.18632/genesandcancer.218

**Published:** 2021-12-27

**Authors:** Panneerselvam Jayabal, Xiuye Ma, Yuzuru Shiio

**Affiliations:** ^1^Greehey Children’s Cancer Research Institute, The University of Texas Health Science Center, San Antonio, TX 78229-3900, USA; ^2^Mays Cancer Center, The University of Texas Health Science Center, San Antonio, TX 78229-3900, USA; ^3^Department of Biochemistry and Structural Biology, The University of Texas Health Science Center, San Antonio, TX 78229-3900, USA

**Keywords:** endogenous retroviruses, Ewing sarcoma, EZH2, interferon response, NELL2

## Abstract

Ewing sarcoma is an aggressive cancer of bone and soft tissue in children. It is characterized by the chromosomal translocation between EWS and an Ets family transcription factor, most commonly FLI1. We recently reported that Ewing sarcoma depends on the autocrine signaling mediated by a cytokine, NELL2. NELL2 signaling stimulates the transcriptional output of EWS-FLI1 through the BAF chromatin remodeling complexes. While studying the impact of NELL2 silencing on Ewing sarcoma, we found that suppression of NELL2 signaling induces the expression of endogenous retroviruses (ERVs) and LINE-1 retrotransposons, an interferon response, and growth arrest. We determined that a histone methyltransferase, EZH2, is the critical downstream target of NELL2 signaling in suppressing ERVs, LINE-1, an interferon response, and growth arrest. We show that EZH2 inhibitors induce ERVs, LINE-1, and an interferon response in a variety of cancer types. These results uncover the role for NELL2–EZH2 signaling in suppressing endogenous virus-like agents and an antiviral response, and suggest the potential utility of EZH2 inhibitors in enhancing anti-tumor immunity.

## INTRODUCTION

Ewing sarcoma is an aggressive bone and soft tissue cancer in children that is characterized by a chromosomal translocation between EWS and an Ets family transcription factor, most commonly FLI1 [1–3]. EWS-FLI1 translocation accounts for 85% of Ewing sarcoma cases. The EWS-FLI-1 gene product functions as an oncogenic transcription factor [1–3], recruiting the BAF chromatin remodeling complexes to its target genes [4].

We recently reported that Ewing sarcoma depends on the autocrine signaling mediated by a cytokine, NELL2 [5]. NELL2 binds to a receptor, Robo3, and stimulates the EWS-FLI1 transcriptional output through inactivation of cdc42, which disassembles and destabilizes the BAF complexes [5]. We identified two populations of cells in Ewing sarcoma, NELL2^high^CD133^high^EWS-FLI1^high^ and NELL2^low^CD133^low^EWS-FLI1^low^, which display phenotypes consistent with high and low NELL2 signaling, respectively [5]. NELL2, CD133, and EWS-FLI1 positively regulate each other and upregulate the BAF complexes and cell proliferation in Ewing sarcoma [5].

About half of human genome is composed of retrotransposons [6], which can copy and paste themselves into different genomic locations through RNA intermediates. Retrotransposons are classified into those that contain long terminal repeats (LTRs) and those that lack LTRs. The former includes endogenous retroviruses (ERVs) while the latter includes long interspersed nuclear elements (LINEs) and short interspersed nuclear elements (SINEs). Retrotransposons are normally silenced by epigenetic mechanisms [7]. Reactivation of retrotransposons by DNA demethylation [8, 9] or during cellular senescence and organismal aging [10] results in an interferon response.

Upon further investigation of the impact of NELL2 signaling on Ewing sarcoma, we found that the suppression of NELL2 signaling induces the expression of endogenous retroviruses (ERVs) and LINE-1 retrotransposons and an interferon response in Ewing sarcoma. We identified EZH2 as a critical downstream target of NELL2 signaling in suppressing ERVs, LINE-1, and an interferon response. Furthermore, we determined that EZH2 inhibitors induce ERVs, LINE-1 and an interferon response in a variety of cancer types. These results uncover the role for NELL2–EZH2 signaling in suppressing ERVs, LINE-1, and an interferon response, and suggest the potential utility of EZH2 inhibitors in enhancing anti-tumor immune responses.

## RESULTS AND DISCUSSION

### Suppression of NELL2 signaling induces an interferon response in Ewing sarcoma

**Figure 1:**
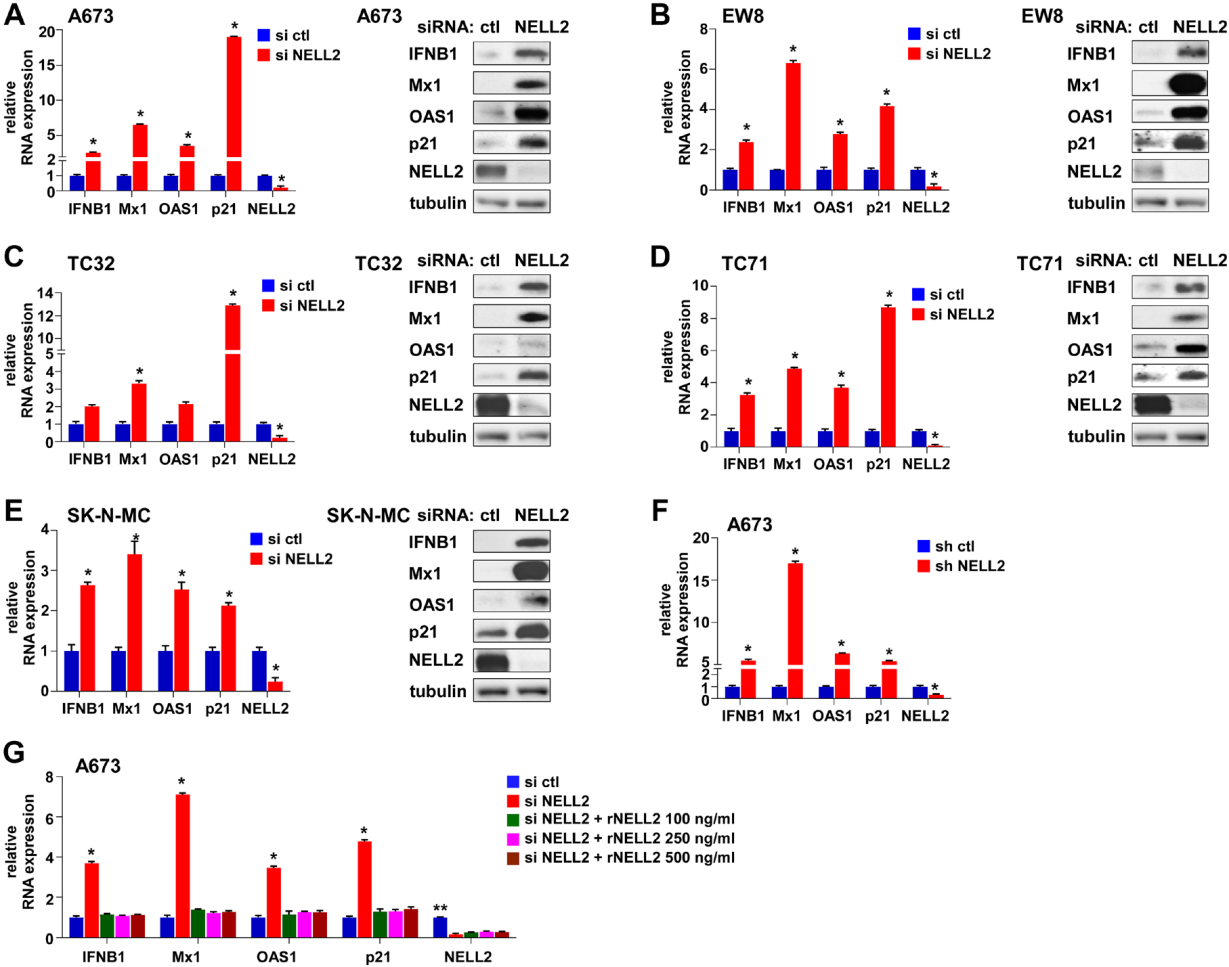
Suppression of NELL2 signaling induces an interferon response in Ewing sarcoma. (**A**–**E**) siRNA-mediated silencing of NELL2 induces an interferon response in Ewing sarcoma cells. Ewing sarcoma cells were transfected with NELL2 siRNA pool or control siRNA pool and the expression of IFNB1, Mx1, OAS1, p21, and NELL2 was analyzed by quantitative RT-PCR (left) and immunoblotting (right). ^*^p < 0.05 compared with control siRNA transfected cells. (A) A673 cells, (B) EW8 cells, (C) TC32 cells, (D) TC71 cells, and (E) SK-N-MC cells. (**F**) shRNA-mediated silencing of NELL2 induces an interferon response in A673 cells. NELL2 was silenced by lentiviral expression of shRNA in A673 cells and the expression of IFNB1, Mx1, OAS1, p21, and NELL2 was analyzed by qRT-PCR. ^*^p < 0.05 compared with control shRNA expressing cells. (**G**) Extracellular NELL2 signals to suppress an interferon response in Ewing sarcoma cells. A673 cells were transfected with NELL2 siRNA pool or control siRNA pool and were left untreated or treated with the indicated concentration of recombinant NELL2 protein for 24 hours. The expression of IFNB1, Mx1, OAS1, p21, and NELL2 was analyzed by qRT-PCR. ^*^p < 0.05 compared with control siRNA transfected cells and with cells transfected with NELL2 siRNA and treated with recombinant NELL2. ^**^p < 0.05 compared with cells transfected with NELL2 siRNA and left untreated or treated with recombinant NELL2.

**Figure 2:**
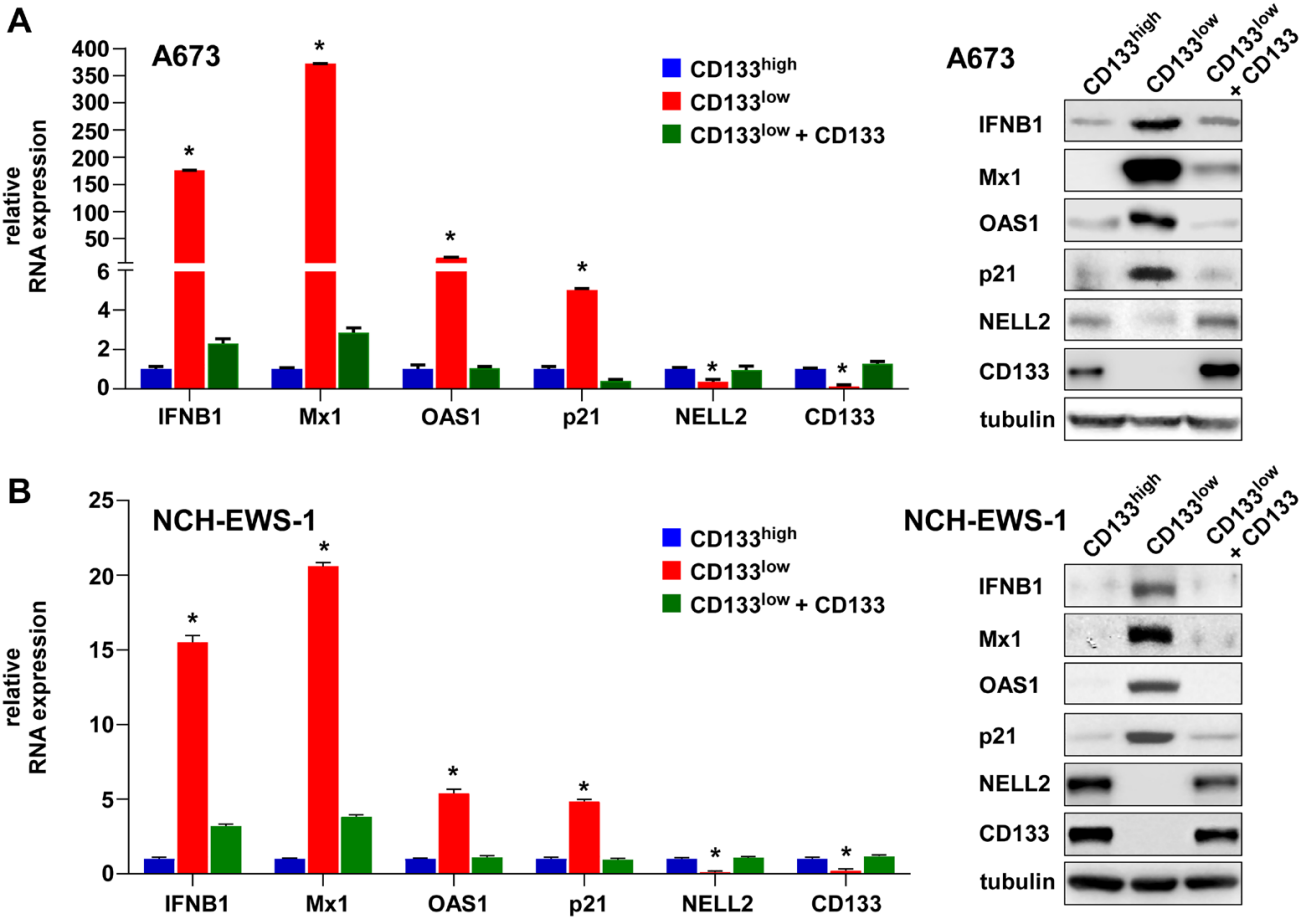
The CD133^low^ population displays an interferon response, which can be suppressed by exogenous CD133. A673 cells (**A**) and NCH-EWS-1 cells (**B**) were incubated with anti-CD133 (AC133) antibody and were sorted into the CD133^high^ and CD133^low^ populations. Part of the CD133^low^ population was infected with CD133-expressing lentivirus and selected with puromycin. The expression of IFNB1, Mx1, OAS1, p21, NELL2, and CD133 was analyzed by qRT-PCR (left) and immunoblotting (right). ^*^p < 0.05 compared with the CD133^high^ population and with the CD133^low^ population expressing exogenous CD133.

We recently reported that the silencing of NELL2 in Ewing sarcoma cells profoundly impairs anchorage-dependent and anchorage-independent growth and xenograft tumorigenicity [5]. Upon further investigation, we discovered that NELL2 silencing by siRNA transfection or lentiviral shRNA expression results in the induction of interferon β1, interferon-stimulated genes (Mx1 and OAS1), and a cyclin-dependent kinase inhibitor p21 (CDKN1A) in Ewing sarcoma cells (Figure 1A–1F), which was suppressed by the addition of recombinant NELL2 protein to the culture medium (Figure 1G). These results suggest that NELL2 signaling suppresses an interferon response in Ewing sarcoma. Ewing sarcoma harbors two populations of cells, NELL2^high^CD133^high^EWS-FLI1^high^ and NELL2^low^CD133^low^EWS-FLI1^low^, which display phenotypes consistent with high and low NELL2 signaling, respectively [5]. Using both an established Ewing sarcoma cell line, A673, and Ewing sarcoma cells dissociated from a patient-derived xenograft tumor (NCH-EWS-1), we found that the CD133^low^ population displays much higher expression levels of interferon β1, Mx1, OAS1, and p21 than the CD133^high^ population (Figure 2A and 2B). We used lentivirus to increase CD133 in the CD133^low^ population to the levels comparable to those of the CD133^high^ population, which restored NELL2 expression (Figure 2; also see Figure 6 in [5]) and suppressed interferon β1, Mx1, OAS1, and p21 (Figure 2A and 2B), further supporting the notion that NELL2 signaling suppresses an interferon response in Ewing sarcoma.

### Suppression of NELL2 signaling induces the expression of endogenous retroviruses (ERVs) and LINE-1 retrotransposons in Ewing sarcoma

**Figure 3:**
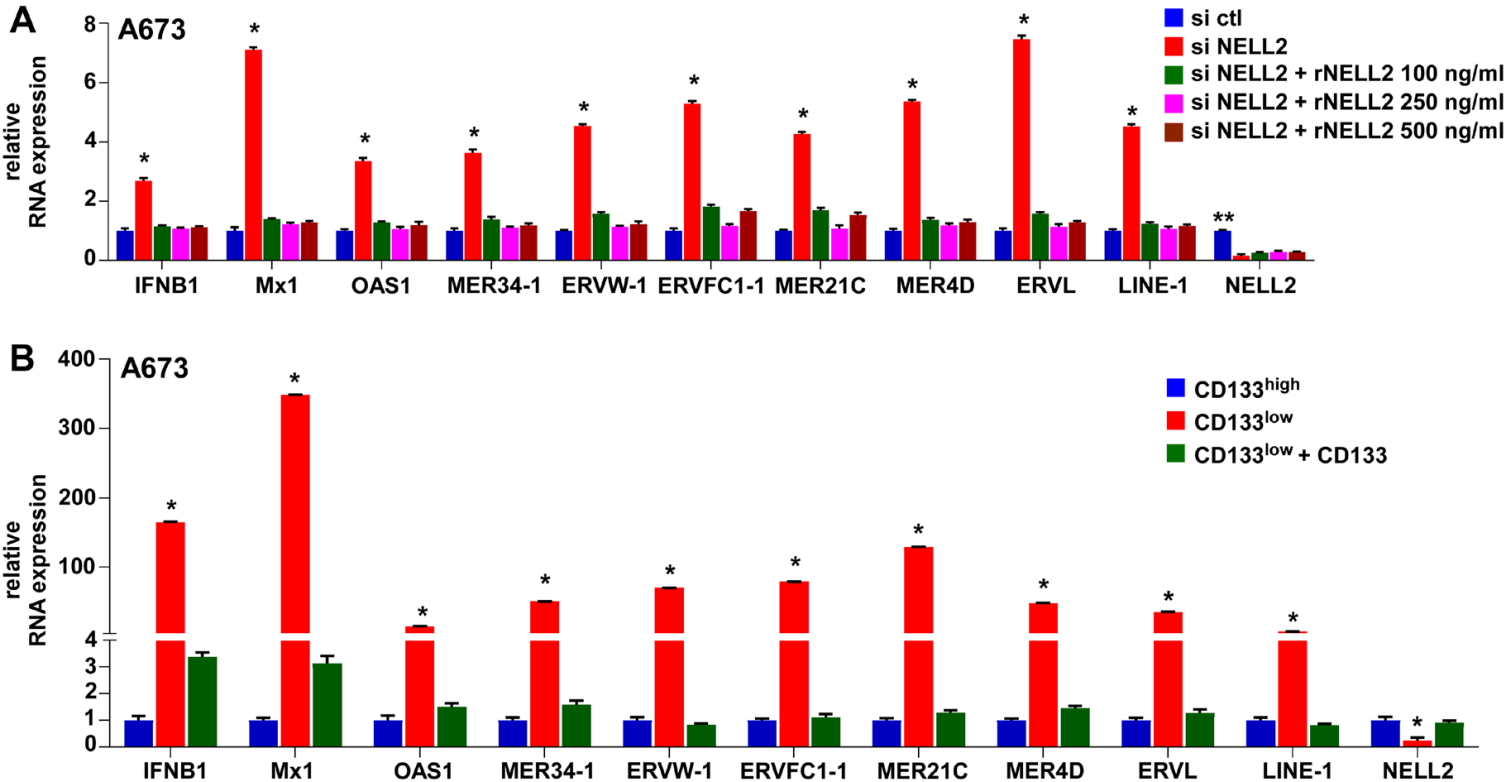
Suppression of NELL2 signaling induces the expression of endogenous retroviruses (ERVs) and LINE-1 retrotransposons in Ewing sarcoma. (**A**) Extracellular NELL2 signals to suppress ERVs and LINE-1 in Ewing sarcoma cells. A673 cells were transfected with NELL2 siRNA pool or control siRNA pool and were left untreated or treated with the indicated concentration of recombinant NELL2 protein for 24 hours. The expression of the indicated genes was analyzed by qRT-PCR. NELL2 silencing induced the expression of ERVs and LINE-1, which was suppressed by recombinant NELL2. ^*^p < 0.05 compared with control siRNA transfected cells and with cells transfected with NELL2 siRNA and treated with recombinant NELL2. ^**^p < 0.05 compared with cells transfected with NELL2 siRNA and left untreated or treated with recombinant NELL2. (**B**) The CD133^low^ population expresses high levels of ERVs and LINE-1, which can be suppressed by exogenous CD133. Cell populations in Figure 2 were analyzed for the expression of the indicated genes by qRT-PCR. ^*^p < 0.05 compared with the CD133^high^ population and with the CD133^low^ population expressing exogenous CD133.

The induction of an interferon response upon suppression of NELL2 signaling in Ewing sarcoma prompted us to search for activation of endogenous virus-like agents. We found that the expression of multiple families of endogenous retroviruses (ERVs) and LINE-1 retrotransposons is induced upon NELL2 silencing, which was suppressed by recombinant NELL2 (Figure 3A), suggesting that extracellular NELL2 signals to suppress ERVs and LINE-1. The CD133^low^ population also displayed much higher expression levels of ERVs and LINE-1 than the CD133^high^ population, which were suppressed by increasing CD133 (Figure 3B). These results suggest that NELL2 signaling suppresses the expression of ERVs and LINE-1 in Ewing sarcoma.

### EZH2 suppresses ERVs, LINE-1, and an interferon response downstream of NELL2 signaling

**Figure 4:**
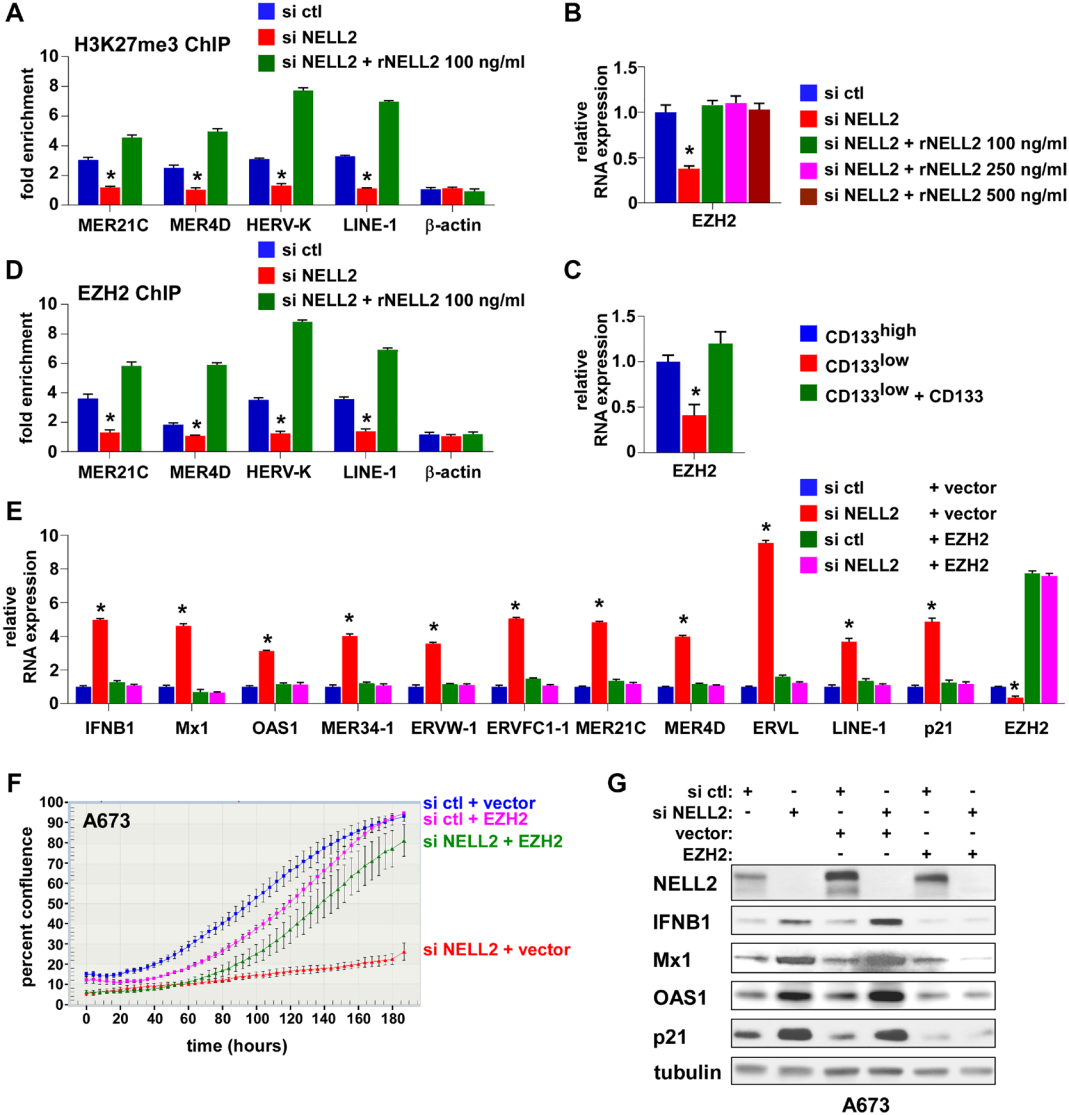
EZH2 suppresses ERVs, LINE-1, and an interferon response downstream of NELL2 signaling. (**A**) Extracellular NELL2 signals to maintain histone H3K27me3 modification in ERVs and LINE-1 in Ewing sarcoma cells. A673 cells were transfected with NELL2 siRNA pool or control siRNA pool and were left untreated or treated with 100 ng/ml of recombinant NELL2 protein for 24 hours. Histone H3K27me3 modification in ERVs and LINE-1 was analyzed by chromatin immunoprecipitation. ^*^p < 0.05 compared with control siRNA transfected cells and with cells transfected with NELL2 siRNA and treated with recombinant NELL2. (**B**) EZH2 expression is regulated by NELL2 signaling in Ewing sarcoma. A673 cells were transfected with NELL2 siRNA pool or control siRNA pool and were left untreated or treated with the indicated concentration of recombinant NELL2 protein for 24 hours. The expression of EZH2 was analyzed by qRT-PCR. NELL2 silencing resulted in reduced EZH2 expression, which was restored by recombinant NELL2. ^*^p < 0.05 compared with control siRNA transfected cells and with cells transfected with NELL2 siRNA and treated with recombinant NELL2. (**C**) The CD133^low^ population displays low EZH2 expression, which was rescued by exogenous CD133. Cell populations in Figure 2 were analyzed for the expression of EZH2 by qRT-PCR. ^*^p < 0.05 compared with the CD133^high^ population and with the CD133^low^ population expressing exogenous CD133. (**D**) NELL2 signaling maintains EZH2 binding to ERVs and LINE-1. A673 cells were transfected with NELL2 siRNA pool or control siRNA pool and were left untreated or treated with 100 ng/ml of recombinant NELL2 protein for 24 hours. The binding of EZH2 to ERVs and LINE-1 was assessed by chromatin immunoprecipitation. NELL2 silencing reduced EZH2 binding to ERVs and LINE-1, which was restored by recombinant NELL2. ^*^p < 0.05 compared with control siRNA transfected cells and with cells transfected with NELL2 siRNA and treated with recombinant NELL2. (**E**) EZH2 suppresses an interferon response induced by NELL2 silencing. A673 cells were transfected with NELL2 siRNA pool or control siRNA pool, followed by transfection of EZH2 or empty vector. The expression of the indicated genes was analyzed by qRT-PCR. ^*^p < 0.05 compared with cells transfected with control siRNA and empty vector and with cells transfected with EZH2. (**F**) EZH2 suppresses growth arrest induced by NELL2 silencing. A673 cells were transfected with NELL2 siRNA pool or control siRNA pool, followed by transfection of EZH2 or empty vector. Cell proliferation was assessed using the IncuCyte live-cell imaging system. (**G**) EZH2 expression does not affect siRNA-mediated NELL2 silencing. A673 cells were transfected with NELL2 siRNA pool or control siRNA pool, followed by transfection of EZH2 or empty vector. The protein levels of NELL2, IFNB1, Mx1, OAS1, and p21 were assessed by immunoblotting. Tubulin serves as a loading control.

ERVs and LINE-1 are normally silenced by epigenetic mechanisms [7]. We found that NELL2 silencing results in dramatically reduced histone H3K27me3 modification in ERVs and LINE-1, which was restored by recombinant NELL2 (Figure 4A). H3K27me3 is the repressive histone modification generated by the Polycomb Repression Complex 2 (PRC2), whose catalytic subunit is EZH2. EZH2 is a transcriptional activation target of EWS-FLI1 [11, 12] and is one of the EWS-FLI1 targets regulated by NELL2 signaling ([5], and Figure 4B and 4C). NELL2 silencing resulted in reduced EZH2 expression, which was restored by recombinant NELL2 (Figure 4B). The CD133^low^ population displayed lower expression levels of EZH2 than the CD133^high^ population, which were increased by increasing CD133 (Figure 4C). These findings raised the possibility that NELL2 signaling stimulates EZH2 expression, leading to H3K27me3 modification and suppression of ERVs and LINE-1. Consistent with this model, NELL2 silencing resulted in reduced EZH2 binding to ERVs and LINE-1, which was restored by recombinant NELL2 (Figure 4D), indicating that NELL2 signaling normally maintains EZH2 binding to ERVs and LINE-1. Furthermore, the induction of ERVs, LINE-1, an interferon response, and growth arrest by NELL2 silencing was suppressed by EZH2 (Figure 4E–4G). These results indicate that EZH2 is the critical downstream target of NELL2 signaling in suppressing ERVs, LINE-1, an interferon response, and growth arrest in Ewing sarcoma.

### EZH2 inhibitors induce an interferon response in a variety of cancers

**Figure 5:**
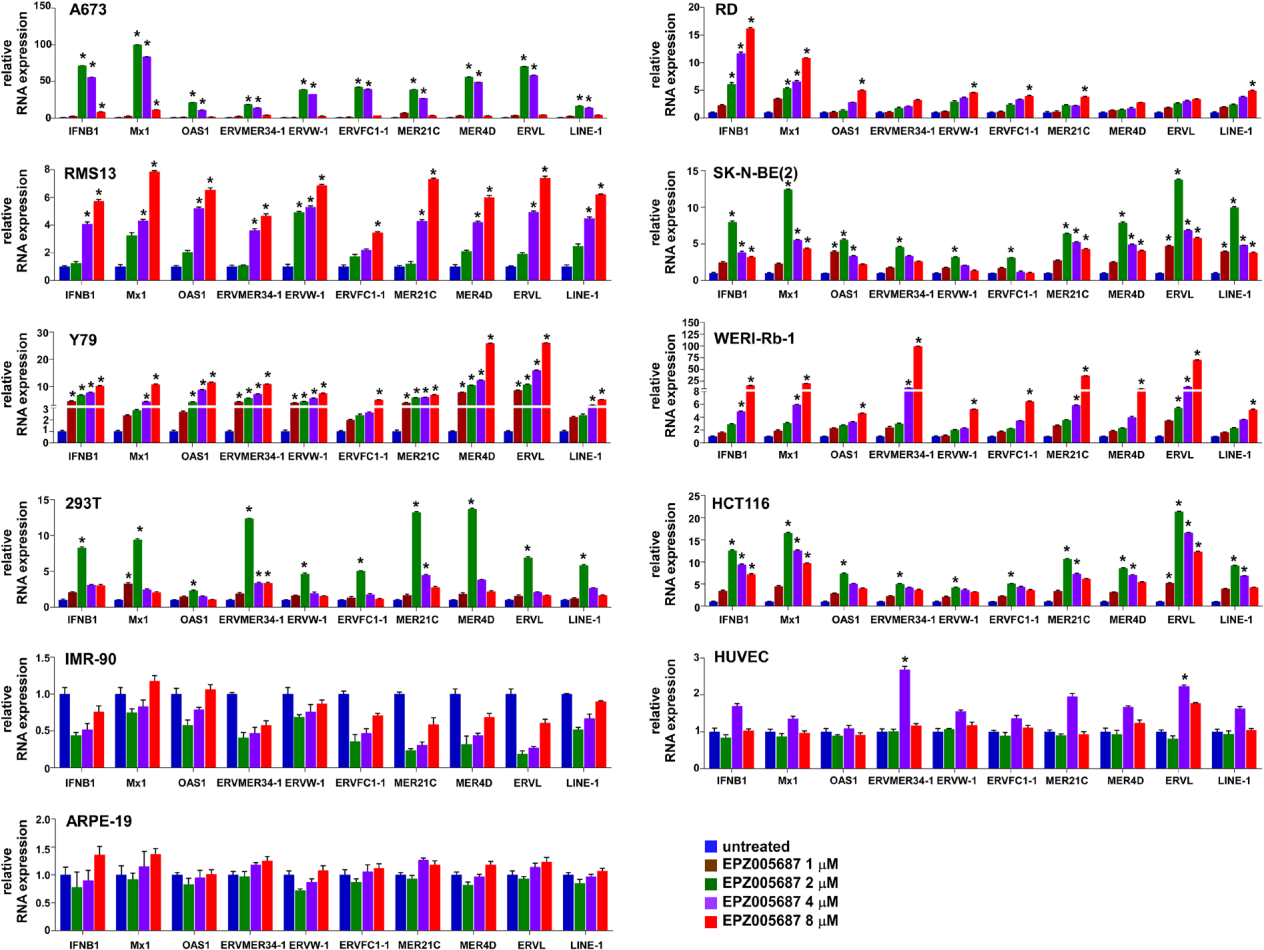
EPZ005687 induces ERVs, LINE-1, and an interferon response in transformed cells. A673, RD, RMS13, SK-N-BE(2), Y79, WERI-Rb-1, 293T, HCT116, IMR-90, human umbilical vein endothelial cells (HUVEC), and ARPE-19 cells were treated with the indicated concentration of EPZ005687 for 72 hours and the expression of the indicated genes was analyzed by qRT-PCR. ^*^p < 0.05 compared with untreated cells.

**Figure 6:**
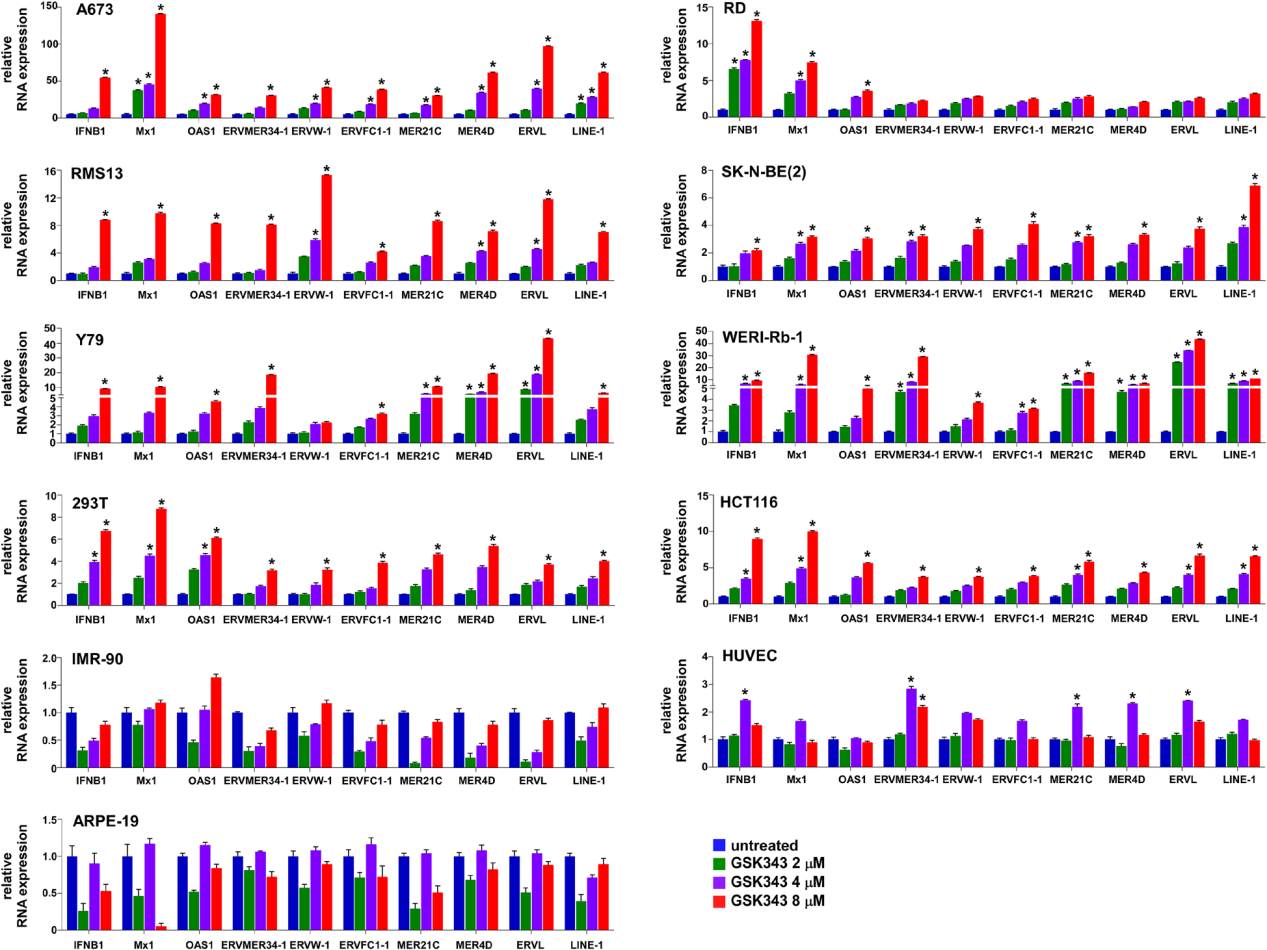
GSK343 induces ERVs, LINE-1, and an interferon response in transformed cells. A673, RD, RMS13, SK-N-BE(2), Y79, WERI-Rb-1, 293T, HCT116, IMR-90, human umbilical vein endothelial cells (HUVEC), and ARPE-19 cells were treated with the indicated concentration of GSK343 for 72 hours and the expression of the indicated genes was analyzed by qRT-PCR. ^*^p < 0.05 compared with untreated cells.

The suppression of endogenous virus-like agents and an interferon response by EZH2 in Ewing sarcoma led us to assess whether EZH2 plays a similar role in other cancer types. The EZH2 inhibitors, EPZ005687 and GSK343, induced the expression of multiple ERVs and LINE-1 and an interferon response in a variety of cancer/transformed cells, including A673 Ewing sarcoma cells, RD and RMS13 rhabdomyosarcoma cells, Y79 and WERI-Rb-1 retinoblastoma cells, SK-N-BE(2) neuroblastoma cells, 293T embryonic kidney cells, and HCT116 colon cancer cells (Figures 5 and 6). Interestingly, however, the EZH2 inhibitors largely did not affect the expression of ERVs, LINE-1 and interferon response genes in non-transformed cells such as IMR-90 primary human fibroblasts, primary human umbilical vein endothelial cells (HUVEC), and ARPE-19 retinal pigment epithelial cells (Figures 5 and 6). These results suggest that EZH2 suppresses ERVs, LINE-1, and an interferon response in a variety of transformed cells, but not in non-transformed cells.

The viral mimicry is emerging as an important mechanism of action for epigenetic cancer therapies. Seminal studies by Roulois et al. and Chiappinelli et al. demonstrated that DNA methylation inhibitors trigger cytotoxic antiviral responses through transcriptional activation of ERVs [8, 9]. The genetic or pharmacological ablation of the histone demethylase LSD1 was also shown to activate the expression of ERVs and induce an interferon response, leading to enhanced anti-tumor immune response [13]. The histone deacetylase inhibitors alone or in combination with DNA methyltransferase inhibitors also induce the expression ERVs [14, 15]. CDK4/6 inhibitors were also shown to suppress DNMT1 and induce the expression of ERVs, leading to an interferon response and enhanced anti-tumor immunity [16]. In the present study, we demonstrated that NELL2 signaling normally suppresses the expression of ERVs and LINE-1, preventing an interferon response in Ewing sarcoma. Inhibiting NELL2 signaling by siRNA/shRNA-mediated silencing of NELL2 robustly induced the expression of ERVs and LINE-1 and an interferon response. Ewing sarcoma tumors usually lack immune and inflammatory cell infiltrates and are considered immunologically “cold” tumors [2]. Targeting NELL2 signaling can not only inhibit tumor growth [5], but also elicit anti-tumor immune response to the immunologically “cold” Ewing sarcoma through the viral mimicry. While the lack of genetically engineered mouse models for Ewing sarcoma [17] is a well-known obstacle to studying anti-Ewing tumor immunity, humanized mouse xenograft models [18, 19] could be developed for Ewing sarcoma, which would allow the assessment of enhancement of anti-tumor immunity by NELL2-EZH2 targeting.

This study also uncovered the key role for EZH2 in suppressing ERVs and LINE-1 and preventing an interferon response in cancers. While we identified this role for EZH2 from the dissection of NELL2 signaling in Ewing sarcoma, EZH2 inhibitors activated ERVs and LINE-1 and induced an interferon response in a variety of cancer types, suggesting the general role for EZH2 in suppressing ERVs, LINE-1, and an interferon response. The role for EZH2 in suppressing ERVs and LINE-1 was reported previously: Ishak et al. demonstrated that EZH2 is recruited to repetitive DNA sequences such as ERVs and LINE-1 through interaction with Rb and E2F1 and that an Rb mutation disrupting E2F1 interaction abrogates EZH2 recruitment to repetitive sequences and results in de-silencing of these sequences [20].

Interestingly, non-transformed cells such as primary fibroblasts, primary umbilical vein endothelial cells, and retinal pigment epithelial cells were refractory to the induction of ERVs, LINE-1, and an interferon response by EZH2 inhibitors (Figures 5 and 6), suggesting that EZH2 inhibitors selectively induce the viral mimicry state in cancer cells. In epigenetically silenced genes, removal of one repressive epigenetic mark (e.g., DNA methylation) often results in accumulation of alternative silencing mark(s) (e.g., histone H3K9me3 or H3K27me3) [21–23], which is termed an “epigenetic switch [24].” Most DNA methylation in mammalian genome is located in the transposable elements [25]. A global loss of DNA methylation is commonly observed in a variety of tumors [26], and many transposable elements lose DNA methylation in tumors [27]. We therefore hypothesize that an epigenetic switch from DNA methylation to EZH2-mediated H3K27me3 modification occurs in some of the transposable elements such as ERVs and LINEs in tumors, leading to tumor-specific induction of the viral mimicry state by EZH2 inhibitors. It will now be important to test the enhancement of anti-tumor immunity by EZH2 inhibitors.

## MATERIALS AND METHODS

### Cell culture

A673, SK-N-MC, 293T, HCT116, and IMR-90 cells were cultured in Dulbecco’s modified Eagle’s medium (DMEM) supplemented with 10% fetal calf serum. EW8, TC71, TC32, RD, RMS13, SK-N-BE(2), and Y79 cells were cultured in RPMI 1640 medium supplemented with 10% fetal calf serum. WERI-Rb-1 cells were cultured in Iscove’s modified Dulbecco’s medium (IMDM) supplemented with 10% fetal bovine serum, 10 μg/ml insulin, and 55 μM β-mercaptoethanol. Human umbilical vein endothelial cells (HUVEC) were cultured in F-12K medium (Kaighn’s Modification of Ham’s F-12 Medium) supplemented with 10% fetal bovine serum. ARPE-19 cells were cultured in Dulbecco’s modified Eagle’s medium/Nutrient Mixture F-12 (DMEM/F-12) supplemented with 10% fetal bovine serum. NCH-EWS-1 cells dissociated from a Ewing sarcoma patient-derived xenograft tumor were cultured in DMEM/F-12 medium supplemented with 10% FBS [5]. A673, SK-N-MC, RD, RMS13, SK-N-BE(2), Y79, WERI-Rb-1, 293T, HCT116, IMR-90, and ARPE-19 cells were from ATCC. TC71 cells were from the Coriell Institute for Medical Research. EW8 and TC32 cells were from Dr. Patrick Grohar. Human umbilical vein endothelial cells were purchased from Lonza. The cell lines were STR-authenticated and were routinely tested for the absence of mycoplasma. Calcium phosphate co-precipitation was used for transfection of 293T cells. Lentiviruses were prepared by transfection in 293T cells following System Biosciences’ protocol and the cells infected with lentiviruses were selected with 2 μg/ml puromycin for 48 hours as described [5, 28]. The target sequences for shRNAs are as follows: NELL2 shRNA, CCTACTTTGAAGGAGAAAGAA, and control shRNA, CCTAAGGTTAAGTCGCCCTCG. The following siRNAs were used: human NELL2 siRNA SMARTpool (M-012185-00; Dharmacon) and Non-Targeting siRNA Pool #2 (D-001206-14-05; Dharmacon). siRNA transfection was performed using Lipofectamine™ RNAiMAX Transfection Reagent (Thermo Fisher). Recombinant NELL2 protein (8946-NL-050) was purchased from R&D Systems. EPZ005687 and GSK343 were purchased from APExBIO Technology.

### Flow cytometry

Cells were trypsinized, washed with FACS wash buffer (PBS, 0.5% BSA, 2 mM EDTA), and incubated with PE-conjugated human CD133/1 antibody (clone AC133, Miltenyi Biotec; 1:100 in FACS wash buffer) for 20 minutes at 4°C. Cells were washed three times with FACS wash buffer and the CD133^high^ and CD133^low^ cell populations were sorted by using BD FACSAria (Becton Dickinson). The FACSDiva 6.1.3 software (Becton Dickinson) was used for sample analysis.

### RNA samples and quantitative real-time PCR

Total cellular RNA was isolated using TRIzol reagent (Invitrogen). Reverse transcription was performed using a High Capacity cDNA Reverse Transcription Kit (Thermo Fisher) as per manufacturer’s instructions. Quantitative PCR was performed using PowerUp SYBR Green Master Mix (Thermo Fisher) on Applied Biosystems ViiA 7 Real-Time PCR System. Each sample was analyzed in triplicate. The following primers were used: NELL2 forward, 5′-GCACAAGCTCTCCTTAGCCAT-3′, NELL2 reverse, 5′-AGGGCTTTTCTACTACCCTTTCA-3′; EZH2 forward, 5′-TGGGAAAGTACACGGGGATA-3′, EZH2 reverse, 5′-TATTGACCAAGGGCATTCAC-3′; CD133 forward, 5′-GACCGACTGAGACCCAACAT-3′, CD133 reverse, 5′-TGGTTTGGCGTTGTACTCTG-3′; IFNB1 forward, 5′-ATGACCAACAAGTGTCTCCTCC-3′, IFNB1 reverse, 5′-GGAATCCAAGCAAGTTGTAGCTC-3′; MX1 forward, 5′-GGTGGTCCCCAGTAATGTGG-3′, MX1 reverse, 5′-CGTCAAGATTCCGATGGTCCT-3′; OAS1 forward, 5′-AGCTTCGTACTGAGTTCGCTC-3′, OAS1 reverse, 5′-CCAGTCAACTGAACCAGGG-3′; ERVMER34-1 forward, 5′-GAATTCAGTGCCACTAAGCAGAC-3′, ERVMER34-1 reverse, 5′-TCGGTATATCCAAGACATGATCC-3′; ERVW-1 forward, 5′-ATGGAGCCCAAGATGCAG.

ERVW-1 reverse, 5′-AGATCGTGGGCTAGCAG-3′; ERVFC1-1 forward, 5′-CTCCATTAGTAGCAGTTCCTCTCC-3′, ERVFC1-1 reverse, 5′-GAGAATAGTGGGACCTGTCCTTT-3′; MER21C forward, 5′-GGAGCTTCCTGATTGGCAGA-3′, MER21C reverse, 5′-ATGTAGGGTGGCAAGCACTG-3′; MER4D forward, 5′-CCCTAAAGAGGCAGGACACC-3′, MER4D reverse, 5′-TCAAGCAATCGTCAACCAGA-3′; ERVL forward, 5′-ATATCCTGCCTGGATGGGGT-3′.

ERVL reverse, 5′-GAGCTTCTTAGTCCTCCTGTGT-3′; LINE-1 forward, 5′-TAAACAAAGCGGCCGGGAA-3′, LINE-1 reverse, 5′-AGAGGTGGAGCCTACAGAGG-3′; p21 forward, 5′-GAGGCCGGGATGAGTTGGGAGGAG-3′, p21 reverse, 5′-CAGCCGGCGTTTGGAGTGGTAGAA-3′; and GAPDH forward, 5′-GGTGTGAACCATGAGAAGTATGA-3′, GAPDH reverse, GAGTCCTTCCACGATACCAAAG.

### Immunoblotting

Fifteen μg of whole-cell lysate was separated by SDS-PAGE and analyzed by immunoblotting as described [5, 28]. The following primary antibodies were used: rabbit monoclonal anti-NELL2 (ab181376, Abcam); rabbit monoclonal anti-CD133 (64326, Cell Signaling Technologies); rabbit monoclonal anti-IFN-β1 (D1D7G) (73671, Cell Signaling Technologies); rabbit monoclonal anti-MX1 (D3W7I) (37849, Cell Signaling Technologies); rabbit monoclonal anti-OAS1 (D1W3A) (14498, Cell Signaling Technologies); rabbit monoclonal anti-p21 (DCS60) (2946, Cell Signaling Technologies); and mouse monoclonal anti-tubulin (DM1A, Thermo Fisher Scientific). The following HRP-conjugated secondary antibodies were used: goat anti-rabbit IgG (7074, Cell Signaling Technologies) and horse anti-mouse IgG (7076, Cell Signaling Technologies).

### Chromatin immunoprecipitation

Chromatin immunoprecipitation (ChIP) was performed as described [5]. Cells were treated with 1% formaldehyde for 10 min at room temperature and cross-linking was quenched by 0.1375 M glycine. Harvested cells were lysed in cell lysis buffer (5 mM PIPES [pH 8.0], 85 mM KCl, 0.5% NP-40) and cell nuclei were pelleted. Cell nuclei were lysed in nuclei lysis buffer (50 mM Tris-Cl [pH 8.0], 10 mM EDTA, 1% SDS, protease inhibitors) and sonicated to generate DNA fragments between 300 and 1000 bp in size. The following antibodies were used for chromatin immunoprecipitation: rabbit polyclonal anti-EZH2 (DPAB-DC595, Creative Diagnostics); control rabbit IgG (ab37415, Abcam); mouse monoclonal anti-H3K27me3 (ab6002, Abcam); and control mouse IgG (ab18413, Abcam). DNA was eluted from immunoprecipitate and input using elution buffer (10 mM Tris-Cl [pH 8.0], 300 mM NaCl, 5 mM EDTA, 1% SDS) supplemented with proteinase K for 2 h at 55°C, followed by overnight incubation at 65°C. DNA in supernatant was purified by phenol-chloroform extraction and was used for qPCR. The PCR primer sequences were as follows:

MER21C LTR forward, 5′-TGGAAGAATACAGGAAACAAGCA-3′, MER21C LTR reverse, 5′-TCCAGAAGGCCCTGGTAACT-3′; MER4D LTR forward, 5′-GCTATCGCGTAGACACATGC-3′, MER4D LTR reverse, 5′-CACATCCCAAGGGCTCTACC-3′; HERV-K LTR forward, 5′-CCCTGGGCAATGGAATGTCTCG-3′, HERV-K LTR reverse, 5′-GCTGCCCGCAGGTCCCACCTC-3′; LINE-1 forward, 5′-ACTGGAAACTCTAAAACGCA-3′, LINE-1 reverse, 5′-GATAATATCCTGCAGAGTGT-3′; β-actin forward, 5′-GCTGTTCCAGGCTCTGTTCC-3′, β-actin reverse, 5′-ATGCTCACACGCCACAACATGC-3′.

### Cell proliferation assays

Cell proliferation was assessed by the IncuCyte live-cell imaging system (Essen BioScience). The IncuCyte system monitors cell proliferation by analyzing the occupied area (% confluence) of cell images over time. At least four fields from four wells were assayed for each experimental condition. The cell seeding density was 2000 cells per well in a 96-well plate. For each assay, biological replicates were performed to confirm the reproducibility of results.

### Statistical analysis

Statistical analyses were performed using one-way ANOVA test in GraphPad Prism Software (version 9.1.2). Data are expressed as mean ± SEM. The results were considered significant when *p* < 0.05.
